# Presumed Transmission of 2 Distinct Monkeypox Virus Variants from Central African Republic to Democratic Republic of the Congo 

**DOI:** 10.3201/eid3010.241118

**Published:** 2024-10

**Authors:** Emmanuel Hasivirwe Vakaniaki, Eddy Kinganda-Lusamaki, Sydney Merritt, Francois Kasongo, Emile Malembi, Lygie Lunyanga, Sylvie Linsuke, Megan Halbrook, Ernest Kalthan, Elisabeth Pukuta, Adrienne Amuri Aziza, Jean Claude Makangara Cigolo, Raphael Lumembe, Gabriel Kabamba, Yvon Anta, Pierrot Bolunza, Innocent Kanda, Raoul Ngazobo, Thierry Kalonji, Justus Nsio, Patricia Matoka, Dieudonné Mwamba, Christian Ngandu, Souradet Y. Shaw, Robert Shongo, Joule Madinga, Yap Boum, Laurens Liesenborghs, Eric Delaporte, Ahidjo Ayouba, Nicola Low, Steve Ahuka Mundeke, Lisa E. Hensley, Jean-Jacques Muyembe Tamfum, Emmanuel Nakoune, Martine Peeters, Nicole A. Hoff, Jason Kindrachuk, Anne W. Rimoin, Placide Mbala-Kingebeni

**Affiliations:** Institut National de Recherche Biomédicale, Kinshasa, Democratic Republic of the Congo (E.H. Vakaniaki, E. Kinganda-Lusamaki, F. Kasongo, L. Lunyanga, S. Linsuke, E. Pukuta, A.A. Aziza, J.C. Makangara Cigolo, R. Lumembe, G. Kabamba, Y. Anta, J. Madinga, S.A. Mundeke, J.-J. Muyembe Tamfum, P. Mbala-Kingebeni);; Institute of Tropical Medicine, Antwerp, Belgium (E.H. Vakaniaki, L. Liesenborghs);; Cliniques Universitaires de Kinshasa, Kinshasa University, Kinshasa (E. Kinganda-Lusamaki, J.C. Makangara Cigolo, R. Lumembe, G. Kabamba, S.A. Mundeke, J.-J. Muyembe Tamfum, P. Mbala-Kingebeni);; Université de Montpellier, French National Research Institute for Sustainable Development, INSERM, Montpellier, France (E. Kinganda-Lusamaki, E. Delaporte, A. Ayouba, M. Peeters);; University of California, Los Angeles, California, USA (S. Merritt, M. Halbrook, N.A. Hoff, A.W. Rimoin);; Hemorrhagic Fevers and Monkeypox Program, Ministry of Health, Kinshasa (E. Malembi, T. Kalonji, R. Shongo);; Ministry of Health and Populations, Bangui, Central African Republic (E. Kalthan);; University of Bern, Bern, Switzerland (J.C. Makangara Cigolo, N. Low);; Provincial Health Division, South Ubangi, Democratic Republic of the Congo (P. Bolunza, I. Kanda);; Mbaya Health Zone, South Ubangi (R. Ngazobo);; National Border Hygiene Program, Ministry of Health, Kinshasa (J. Nsio, P. Matoka);; National Institute of Public Health, Ministry of Health, Kinshasa (D. Mwamba, C. Ngandu);; University of Manitoba, Winnipeg, Manitoba, Canada (S.Y. Shaw, J. Kindrachuk);; Pasteur Institute of Bangui, Bangui, Central African Republic (Y. Boum, E. Nakoune);; USDA Agricultural Research Service, Manhattan, Kansas, USA (L.E. Hensley)

**Keywords:** mpox, monkeypox virus, viruses, zoonoses, sexually transmitted infections, Democratic Republic of the Congo, Central African Republic, surveillance, cross-border transmission, subclade Ia, MPXV

## Abstract

We linked 4 mpox cases in South Ubangi, Democratic Republic of the Congo, to transboundary transmission from Central African Republic. Viral genome sequencing demonstrated that the monkeypox virus sequences belonged to distinct clusters of subclade Ia. This finding demonstrates the borderless nature of mpox and highlights the need for vigilant regional surveillance.

Mpox is a zoonotic viral infectious disease first identified in humans in the Democratic Republic of the Congo (DRC) in 1970 (*1*). Monkeypox virus (MPXV) is endemic to forested regions of Central and West Africa (*2*) and is subclassified into clade I and clade II; subclade IIb was responsible for a global epidemic in 2022 (*3*). We have recommended subdivision of clade I into subclades Ia and Ib, where subclade Ib is associated with sustained human-to-human transmission in the DRC (*4*). Clade I is endemic to Central Africa, particularly in the DRC, and infections are associated with greater disease severity than for clade II infection (*5*,*6*). Zoonotic spillover has been the primary driver of clade I MPXV infections; however, sustained human-to-human transmission is increasing, and spread of clade I MPXV through sexual contact has been reported in multiple regions of the DRC (*4*,*7*).

Among mpox-endemic regions, the DRC has been the most severely affected. The number of reported cases has increased since the cessation of smallpox vaccination campaigns in 1980 (*8*). This increase accelerated from 2022 onward (*9*), including into regions with no previously reported cases (*10*). Cross-border travel has been demonstrated as a factor in transmission of clade II MPXV, but less is known for clade I cases in regional settings. Given the recent geographic expansion for clade I MPXV and increasing observation of sustained human-to-human transmission, considerable concerns exist about geographic expansion of mpox through cross-border transmission (*10*).

The Central African Republic (CAR) shares a >1,000-mile border with the DRC. CAR has reported 40 confirmed mpox outbreaks (95 suspected cases during 2001–2021), increasing from 0–2 annually during 2001–2017 to 9 annually since 2018 (*11*). Most of those outbreaks occurred in 2 regions along the border with the DRC. Here, we report suspected cross-border transmission of mpox between CAR and the DRC in 2023 and potential links to MPXV circulation in DRC.

## The Study

The South Ubangi Provincial Health Division in the DRC issued an alert for a suspected mpox case in January 2023 after the death of a fisherman displaying respiratory distress and skin lesions in Mbaya Health Zone. South Ubangi directly borders CAR, separated by the Ubangi River. The National Programme for Control of Mpox and Viral Hemorrhagic Fevers and the National Institute for Biomedical Research deployed a multidisciplinary team to investigate the case with the Provincial Health Division. As part of the investigation, the national and provincial health teams conducted supplemental training sessions to raise awareness of the clinical signs and transmission modes for mpox.

**Figure 1:**
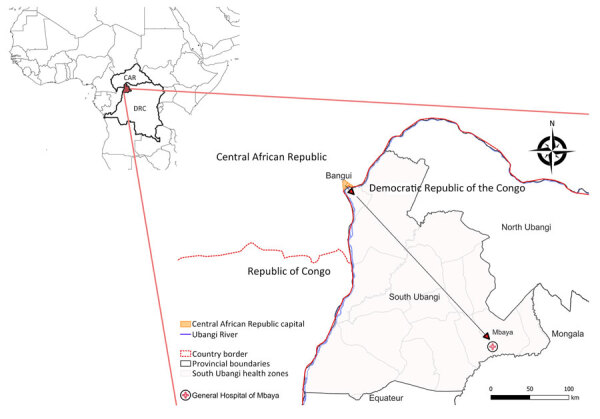
Possible transmission route of monkeypox virus between Bangui, Central African Republic, and Mbaya Health Zone, South Ubangi Province, Democratic Republic of the Congo, January 2023. Inset map shows location of countries and study area within Africa. Created with BioRender.com.

The index case was an adult man residing in Bangui, CAR, who moved frequently between CAR and the DRC by the Ubangi River (cluster 1, case 1: C1) (Figure 1). After suspected exposure to wildlife meat and the potential consumption of rodents in CAR in early January 2023, symptom onset began shortly after (day 0); symptoms were fever, headache, chills, and cutaneous rash. After traveling to the DRC, he was admitted to Mbaya General Reference Hospital 2 days later, where he died on day 9 after symptom onset, after experiencing respiratory distress. No samples or information on his immune status were collected from this probable mpox case-patient.

We identified 15 contacts of the patient in Mbaya Health Zone, consisting of 5 healthcare workers and 10 close contacts (4 close friends and 6 family contacts). The investigation revealed a cluster of 3 symptomatic family contacts. Of those, 2 contacts had samples taken, and both tested positive for MPXV by PCR; those contacts were the adult female partner of C1 (C2) and their child (C3). A second child of C1 and C2 (C4) was treated locally for suspected mpox using traditional methods and was not investigated further. No suspected mpox cases were identified among family or close contacts of C1 in Bangui.

**Figure 2:**
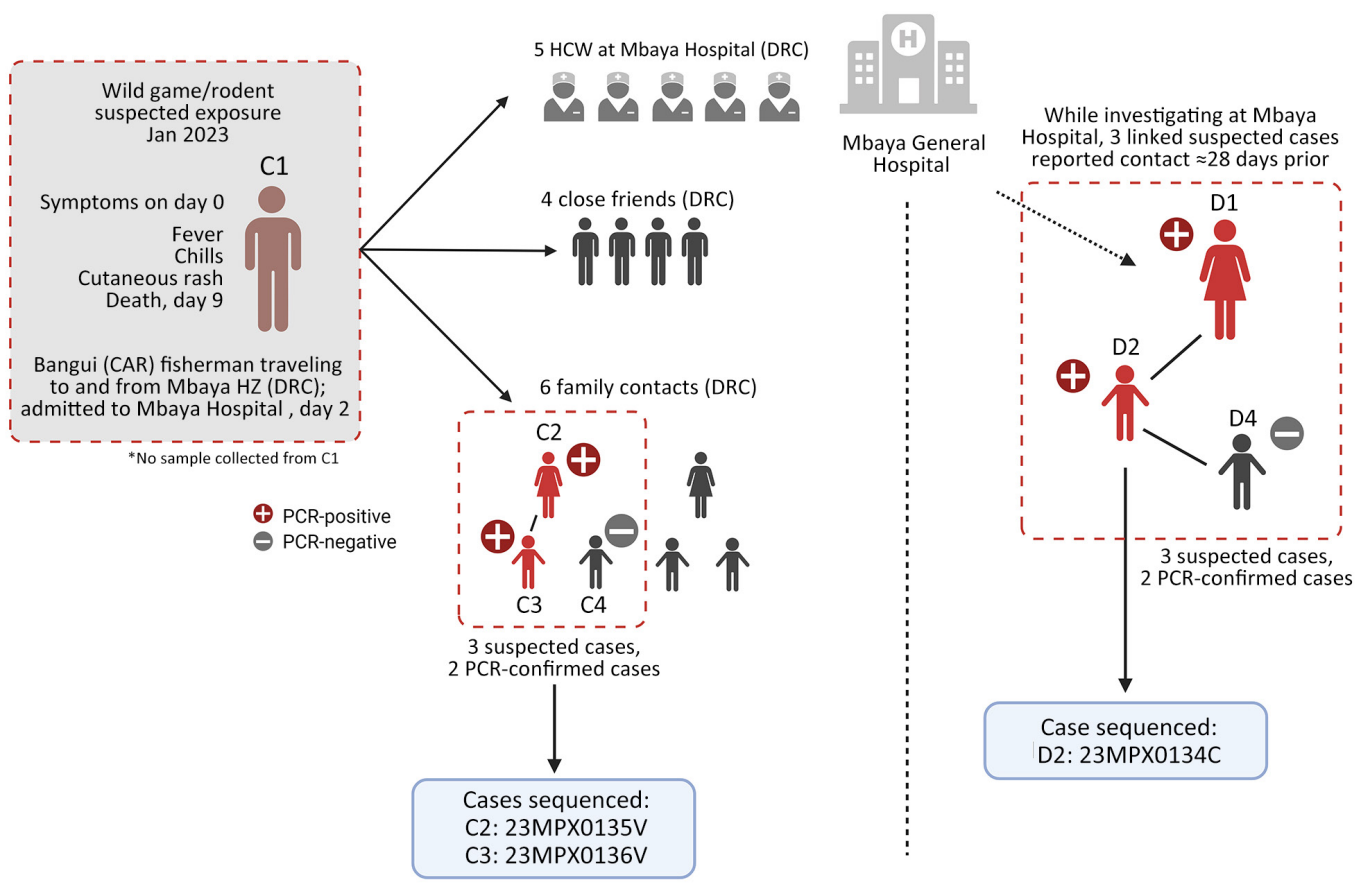
Possible transmission chain of monkeypox virus from CAR to the DRC and second identified chain at Mbaya General Reference Hospital, Mbaya, DRC. CAR, Central African Republic; DRC, Democratic Republic of the Congo; HCW, healthcare workers; HZ, health zone. Created with BioRender.com.

**Figure 3:**
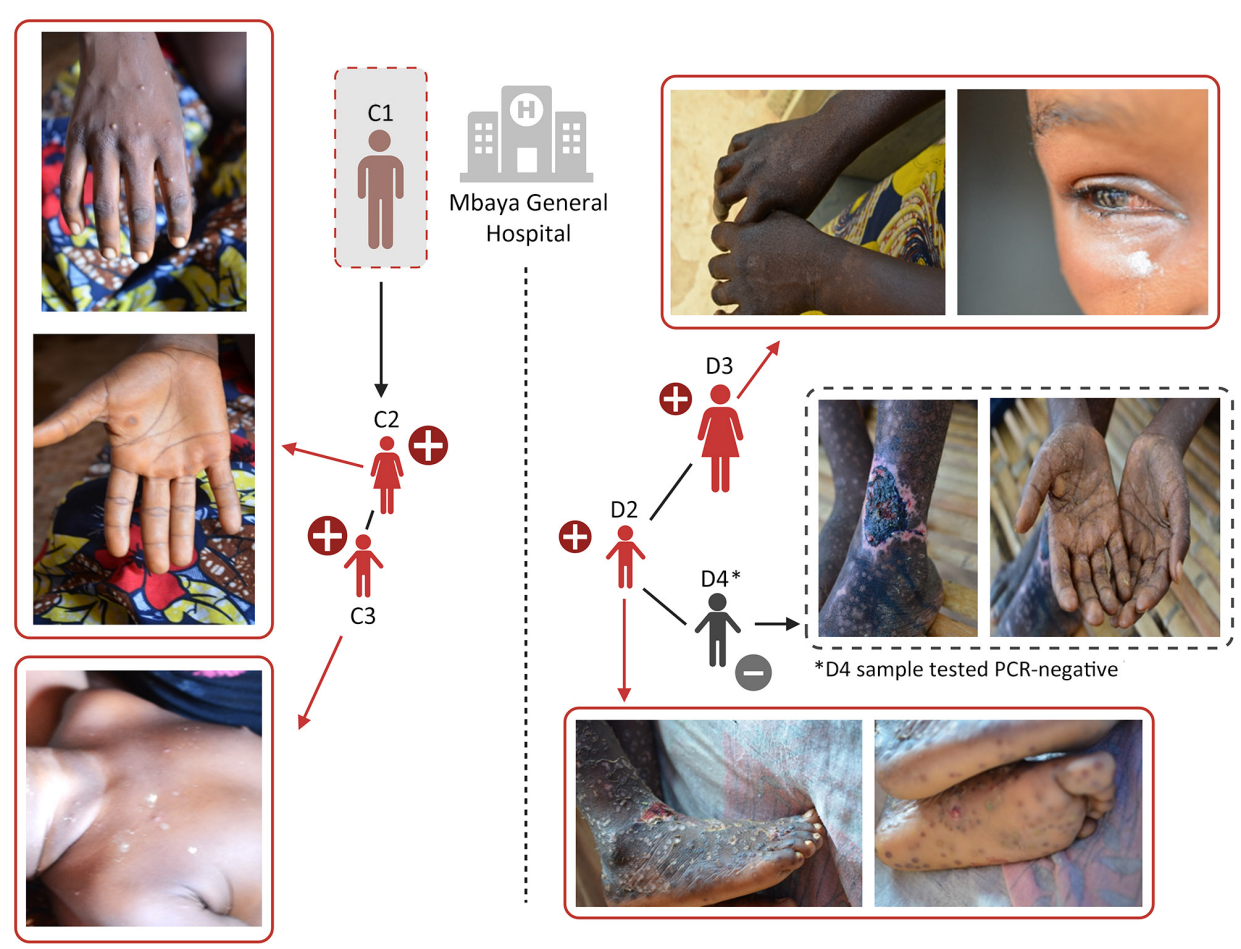
Clinical manifestations of suspected mpox from 2 mother-child pairs in study of presumed transmission of monkeypox virus variants from Central African Republic to Democratic Republic of the Congo. One mother-child pair was linked to the deceased fisherman in Mbaya Health Zone, and the other pair were identified at Mbaya Hospital, Mbaya, Democratic Republic of the Congo. Created with BioRender.com.

During the investigation, we identified a distinct second mpox cluster, which likely originated from a different source. We identified 3 suspected mpox cases in the same hospital at the same time as the first cluster that had no known epidemiologic link. An adult woman (cluster 2, case 1: D1) was hospitalized for nonspecific signs of mpox at the same time as C1 and reported contact with a child with mpox symptoms ≈28 days before. The 2 additional suspected cases were the child of D1 (D2), in whom mpox was subsequently confirmed by PCR, and a contact of D2 (D3), who tested negative by PCR (Figure 2). Confirmed mpox clinical symptoms included multiple pustular and papular lesions (C2); disseminated pustules (C3); discreet lacrimation in the left eye and postinflammatory hyperpigmentation (D1); and hyperpigmented, disseminated macules and steep-edged ulcerations (D2) (Figure 3).

**Figure 4:**
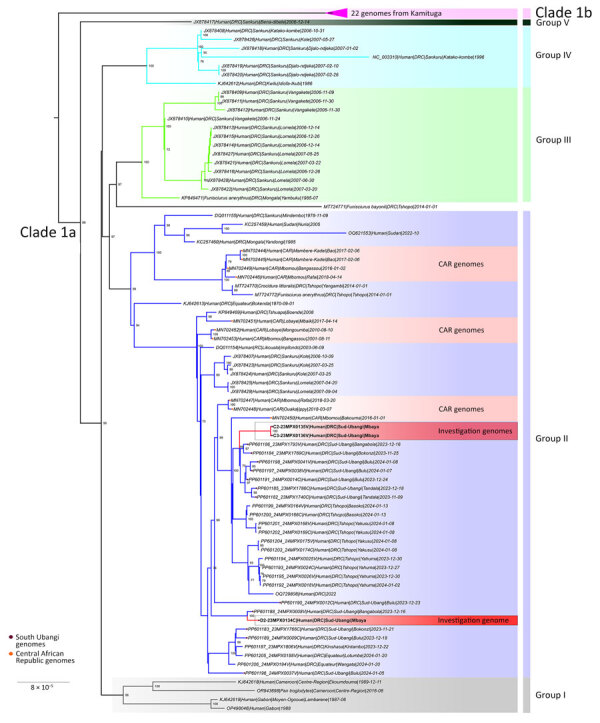
Phylogenetic analysis of monkeypox virus (MPXV) sequences from Mbaya Health Zone in study of presumed transmission of MPXV variants from Central African Republic to Democratic Republic of the Congo. Phylogenetic analysis of MPXV genome sequences are from samples described in this study and clade I MPXV sequences from Central Africa. Bootstrap support values are shown at branch points. DNA was extracted at the National Institute for Biomedical Research using a QIAGEN DNA Mini Kit (https://www.qiagen.com) from blood samples and subsequently screened for MPXV with an orthopoxvirus-specific real-time PCR assay. Whole-genome sequencing was attempted on samples from the index case by next-generation sequencing. Library preparation was performed using Illumina DNA Prep with Enrichment (https://www.illumina.com), and libraries were enriched for MPXV using biotinylated custom probes synthesized by Twist Biosciences (https://www.twistbioscience.com). Note that 23MPX0134C(D2), 23MPX0135V(C2), and 23MPX0136V(C3) are samples from crust or vesicles from separate individuals. Sequences can be accessed with the following GISAID (https://www.gisaid.org) accession nos.: EPI_ISL_19287107 (D2), EPI_ISL_19287108 (C2), EPI_ISL_19287109 (C3). Scale bar indicates number of substitutions per site.

We subsequently performed viral genome sequencing on 3 PCR-positive samples: 2 from the first mpox cluster (C2 and C3) and 1 from the second cluster (D2) (Figure 4). Phylogenetic analysis included 98 clade I MPXV previously published genomes. We generated the phylogenetic tree by maximum-likelihood using the Kimura 3-parameter model with unequal base frequencies plus gamma distribution plus invariable sites (*12*,*13*). The 3 sequences, all clade Ia, clustered with MPXV genome sequences isolated from CAR. The genetic distance between the new cases and the date between the CAR and DRC samples are too long to conclude direct links between the 2 countries. Nevertheless, the new samples from South-Ubangi clustered in the same subgroup (group II) according to previous classification of MPXV sequences in clade Ia (*14*).

Further, the National Programme for Control of Mpox and Viral Hemorrhagic Fevers investigated 109 suspected mpox cases from 15 health zones in South Ubangi during January–November 2023. Case-patients were predominantly male (60 male and 46 female; data were unavailable for 3 cases); the average age was 21 years (range 35 months–63 years). Of those, 61 cases (56%) were confirmed positive by PCR at National Institute for Biomedical Research. Three of the 46 mpox-negative cases (6%) were confirmed as positive for varicella zoster virus.

## Conclusions

This investigation describes 2 distinct clusters of mpox cases in Mbaya Health Zone in South Ubangi Province in the DRC, presumably resulting from 2 different introductions. We identified epidemiologic links to cross-border travel from CAR for the first cluster and identified genomic links with historic CAR cases for both clusters. MPXV transmission in the DRC is currently driven by both zoonotic and human-to-human contact, thus increasing the complexity of containment and mitigation efforts. Wider regional and international expansion of mpox, specifically, the concentration of MPXV outbreaks in CAR and the DRC along the Ubangi River, recent sustained human-to-human transmission, resource limitations for identifying and treating mpox, disease stigma, population displacement because of conflict, and transient cross-border transit are serious concerns.

In August 2022, a first regional meeting involving 6 neighboring countries of Central and West Africa was held in Kinshasa, the DRC, to establish a regional mpox surveillance network, the Mpox Threat Reduction Network. Through this network, multiple teams including experts from the Health Ministries of the DRC and CAR were able to communicate rapidly on suspected cases crossing borders in South Ubangi while also reporting to International Health Regulations focal points. Considering the relatively porous borders in the Central African region and continuing increases in reported mpox cases, documenting transboundary mpox transmission is critical. This investigation highlights the importance of long-term collaborative partnerships for sustained mpox surveillance and containment in endemic regions.
